# Many ways to make darker flies: Intra‐ and interspecific variation in *Drosophila* body pigmentation components

**DOI:** 10.1002/ece3.7646

**Published:** 2021-05-25

**Authors:** Elvira Lafuente, Filipa Alves, Jessica G. King, Carolina M. Peralta, Patrícia Beldade

**Affiliations:** ^1^ Instituto Gulbenkian de Ciência Oeiras Portugal; ^2^ CE3C: Centre for Ecology, Evolution, and Environmental Changes, Faculty of Sciences University of Lisbon Lisbon Portugal; ^3^ Present address: Swiss Federal Institute of Aquatic Science and Technology Department of Aquatic Ecology Dübendorf Switzerland; ^4^ Present address: Institute of Evolutionary Biology School of Biological Sciences University of Edinburgh Edinburgh UK; ^5^ Present address: Max Planck Institute for Evolutionary Biology Plön Germany

**Keywords:** decomposing phenotypes, developmental plasticity, genetic and environmental variance, quantitative phenotyping, thermal plasticity

## Abstract

Body pigmentation is an evolutionarily diversified and ecologically relevant trait with substantial variation within and between species, and important roles in animal survival and reproduction. Insect pigmentation, in particular, provides some of the most compelling examples of adaptive evolution, including its ecological significance and genetic bases. Pigmentation includes multiple aspects of color and color pattern that may vary more or less independently, and can be under different selective pressures. We decompose *Drosophila* thorax and abdominal pigmentation, a valuable eco‐evo‐devo model, into distinct measurable traits related to color and color pattern. We investigate intra‐ and interspecific variation for those traits and assess its different sources. For each body part, we measured overall darkness, as well as four other pigmentation properties distinguishing between background color and color of the darker pattern elements that decorate each body part. By focusing on two standard *D. melanogaster* laboratory populations, we show that pigmentation components vary and covary in distinct manners depending on sex, genetic background, and temperature during development. Studying three natural populations of *D. melanogaster* along a latitudinal cline and five other *Drosophila* species, we then show that evolution of lighter or darker bodies can be achieved by changing distinct component traits. Our results paint a much more complex picture of body pigmentation variation than previous studies could uncover, including patterns of sexual dimorphism, thermal plasticity, and interspecific diversity. These findings underscore the value of detailed quantitative phenotyping and analysis of different sources of variation for a better understanding of phenotypic variation and diversification, and the ecological pressures and genetic mechanisms underlying them.

## Graphical abstract

We characterize genetic and environmental sources of intra‐ and interspecies variation in distinct quantitative properties of *Drosophila* body pigmentation, of interest in eco‐evo‐devo studies.

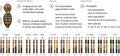

## 1 INTRODUCTION

Diversity in body coloration provides some of the most compelling examples of adaptive evolution. Insect coloration, in particular, includes text book cases such as industrial melanism (e.g., Cook & Saccheri, 2013; van't Hof et al., 2011), mimicry (e.g., Mallet & Joron, 1999; Nadeau, 2016), and clinal variation (e.g., Bastide et al., 2014; Endler et al., 2016; Telonis‐Scott et al., 2011; Wittkopp et al., 2011). Studies in different species have illustrated the ecological significance of variation in body pigmentation, including visual communication between individuals of the same (e.g., mate attraction and mate choice; e.g., Guillermo‐Ferreira et al., 2014; Wiernasz, 1995) or of different species (e.g., predator avoidance via camouflage or aposematism; e.g., van Bergen & Beldade, 2019; Futahashi & Fujiwara, 2008; Reichstein et al., 1968), as well as thermoregulation (e.g., Rajpurohit et al., 2008; Sibilia et al., 2018). Moreover, insect pigmentation is tightly associated with various other traits that are closely related to fitness (see Mckinnon & Pierotti, 2010; Wittkopp & Beldade, 2009). The diversity of insect pigmentation across species, populations, sexes, and individuals of the same sex has been the focus of many eco‐evo‐devo studies, providing key insight into the genetic basis of phenotypic variation (e.g., Futahashi & Fujiwara, 2008; Massey & Wittkopp, 2016; Miyagi et al., 2015; Orteu & Jiggins, 2020; Pool & Aquadro, 2007; Zhang et al., 2017) and exploring important phenomena such as developmental plasticity (e.g., Monteiro et al., 2015; Shearer et al., 2016; Solensky & Larkin, 2003), the origin of novelty (e.g., Shirai et al., 2012; Vargas‐Lowman et al., 2019), and evolutionary constraints (e.g., Allen et al., 2008; Beldade, Brakefield, et al., 2002; Beldade, Koops, et al., 2002).

Variation in body pigmentation can arise from differences in color and/or in the spatial arrangement of colors into specific patterns. These two aspects are believed to rely on largely distinct classes of genes involved in pigmentation development: those encoding the enzymes responsible for pigment synthesis, and those encoding the transcription factors regulating the expression of those enzymes at the appropriate time and location (see True, 2003; Wittkopp & Beldade, 2009; Wittkopp et al., 2003). Changes in genes associated with each of these steps can result in changes in pigmentation between individuals and between body parts (e.g., Wittkopp et al., 2002). In this respect, body pigmentation can be thought of as a multidimensional trait, made up of several components representing aspects of actual color and of color pattern, which might vary between body parts and develop and evolve more or less independently. This has been explored in studies focusing on specific color pattern elements, including on butterfly wings (reviews in Beldade & Peralta, 2017; Monteiro, 2015; Nijhout, 2001), as well as on fly wings and abdomens (e.g., Jeong et al., 2006; Werner et al., 2010). Yet, rarely do studies of body pigmentation variation combine quantitative analysis of multiple color and color pattern traits.

Studies of *Drosophila* body and wing pigmentation have provided very valuable insight about the genetic and environmental bases of variation between species, populations of the same species, and individuals of the same population (e.g., Gibert et al., 2007; Hollocher et al., 2000; Massey & Wittkopp, 2016; Pool & Aquadro, 2007; Wittkopp et al., 2003). These studies characterized effects of environmental factors, such as nutrition (e.g., Shakhmantsir et al., 2014) and temperature (e.g., David et al., 1990), as well as allelic variants of both subtle (e.g., Bastide et al., 2013) and large phenotypic effect (e.g., Carbone et al., 2005). Variation in *Drosophila* pigmentation has been associated with clinal and seasonal variation in desiccation resistance, thermoregulation, and UV protection (e.g., Matute & Harris, 2013; Parkash et al., 2014; Rajpurohit et al., 2008) and shown to correlate with other traits, such as reproductive success, behavior, and immunity (e.g., Dombeck & Jaenike, 2004; Massey et al., 2019; Takahashi, 2013). While studies of *Drosophila* pigmentation have included focus on different body parts (e.g., trident on thorax, e.g., David et al., 1985; melanic patches on wings, e.g., True et al., 1999; dark bands of abdominal segments, e.g., Dembeck et al., 2015), these studies typically analyze single and often qualitative properties of pigmentation (but see, e.g., Saleh Ziabari & Shingleton, 2017). Indeed, the detail in quantitative phenotyping of *Drosophila* pigmentation does not match the sophistication of the analysis of its genetic and developmental bases. This is not unique to *Drosophila* pigmentation; the need for more attention to be given to phenotyping has been called for repeatedly (Deans et al., 2015; Gerlai, 2002; Houle et al., 2010; Kühl & Burghardt, 2013; Laughlin & Messier, 2015).

Here, we provide a detailed analysis of patterns and sources of intra‐ and interspecific variation in body pigmentation in *Drosophila*, considering aspects of both color and color pattern. For that, we quantify five traits encompassing aspects of color and color pattern of abdomen and thorax pigmentation in *Drosophila* adults. We investigate how each of these pigmentation components and the associations between them differ between genotypes and developmental temperatures, within and across species. We show that different pigmentation components can vary rather independently and that fly bodies can be made lighter or darker by changing distinct pigmentation components. We discuss our results in the context of the potential for evolutionary diversification of pigmentation.

## 2 MATERIAL AND METHODS

### 2.1 Fly stocks and experimental design

In this study, we used a total of 23 *Drosophila* fly stocks: two *D. melanogaster* laboratory strains CantonS (CanS) and OregonR (OreR), five different isogenic lines of *D. melanogaster* from each of three geographical locations (i.e., Finland, Austria, and Spain), two strains of *D. simulans* (*D.sim* A and *D.sim* B), one stock of *D. malerkotliana,* one stock of *D. repleta*, one stock of *D. mojavensis baja,* and one stock of *D. mojavensis mojavensis*. *D. melanogaster* strains (CanS and OreR) and *Drosophila* species *D. simulans, D. malerkotliana, D. repleta*, *D. mojavensis baja,* and *D. mojavensis mojavensis* were obtained from C. Mirth's laboratory. *D. melanogaster* populations from Finland (Akaa; 61.1, 23.52; collected in July 2015), Austria (Mauternbach; 48.38, 15.57; collected in July 2016), and Spain (Tomelloso; 39.16, 3.02; collected in September 2015) were obtained from E. Sucena's laboratory and collected by members of the *European Drosophila Population Genomics Consortium* (*DrosEu*; http://droseu.net). From the species used in this study, *D. melanogaster, D. simulans,* and *D. malerkotliana* belong to the melanogaster group, which originally inhabited tropical climates, though they have become cosmopolitan species. In contrast, *D. mojavensis and D. repleta* belong to the repleta group, which inhabits desert climates. All stocks were maintained in molasses food (45 g molasses, 75 g sugar, 70 g cornmeal, 20 g yeast extract, 10 g agar, 1,100 ml water, and 25 ml of Nipagin 10%). All stocks were kept at 25°C, 12:12‐hr light–dark cycles. For the experiments, we performed overnight egg‐laying from ~20 females of each stock in vials with ad libitum molasses food. Eggs were then placed at either 17°C or 28°C throughout development. We controlled the population density by keeping between 20 and 40 eggs per vial.

For the experiment of the windows of sensitivity for pigmentation, we exposed developing flies to 17°C or 28°C during one window of development while they were kept at 23°C for the remaining stages. We tested four different treatments at 17°C and at 28°C: *T* (flies always kept at constant temperature), *L* (late larval development; staging done by using traqueal and mouth hook morphology), *p* (only early pupal period; from white pupa to the onset of eye pigmentation), *P* (only late pupal period; from the onset of eye pigmentation until adult eclosion).

### 2.2 Phenotyping pigmentation components

**FIGURE 1:**
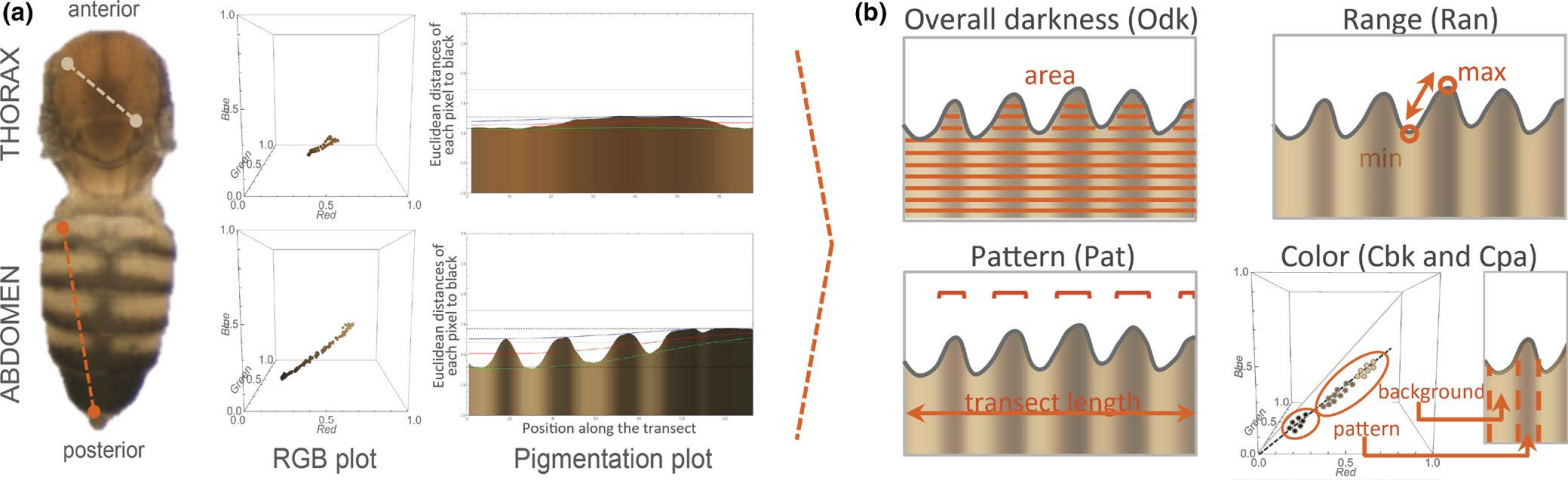
Quantitative analysis of body pigmentation. (a) Thorax and abdomen from female of *D. melanogaster* OreR reared at 17°C showing the body landmarks used to draw the transects. For each pixel in the transect, we extracted RGB values that are represented in the RGB plots (cubes on the right side of each transect). By calculating the distance between each of those pixels to the black, we converted the RGB vectors into two‐dimensional information and represented the distance of each pixel (*y*‐axis) from the anterior to the posterior extremes of the transect (*x*‐axis) (plots on the right side). (b) Diagram showing the different pigmentation traits. Overall darkness (Odk), difference between lightest and darkest color (Ran), relative length of dark “ornamental” pattern (Pat), color of background (Cbk), and color of pattern “ornamental” elements (Cpa) (see Materials and Methods)

Adult flies (8–10 days after eclosion) were placed in 2‐ml microcentrifuge tubes and frozen in liquid nitrogen. The tubes were shaken immediately after submersion in liquid nitrogen to remove wings, legs, and bristles. Headless bodies of flies were then mounted on 3% agarose in Petri dishes, dorsal side up, and covered with water to avoid specular reflection of light upon imaging. Images containing 10–20 flies were collected with a LeicaDMLB2 stereoscope and a Nikon E400 camera under controlled conditions of illumination and white‐balance adjustment. Images were later processed with a set of custom‐made interactive Mathematica notebooks (Wolfram Research, Inc., Mathematica, version 10.2, Champaign, IL, 2015) to extract pigmentation measurements. For this purpose, two transects were defined on each fly, one in the thorax and one in the abdomen, using morphological landmarks (as shown in Figure 1). To minimize image noise, for each pixel position along the transect line we calculated the mean RGB (red, green, blue) values of the closest five pixels located on a small perpendicular line centered on the transect. For abdominal transects, when necessary, we removed the sections corresponding to the membranous tissue that occasionally is visible between abdominal segments. The few transects that were drawn over debris particles were excluded from the analysis, as pigmentation measurements could not be accurately extracted.

**FIGURE 2:**
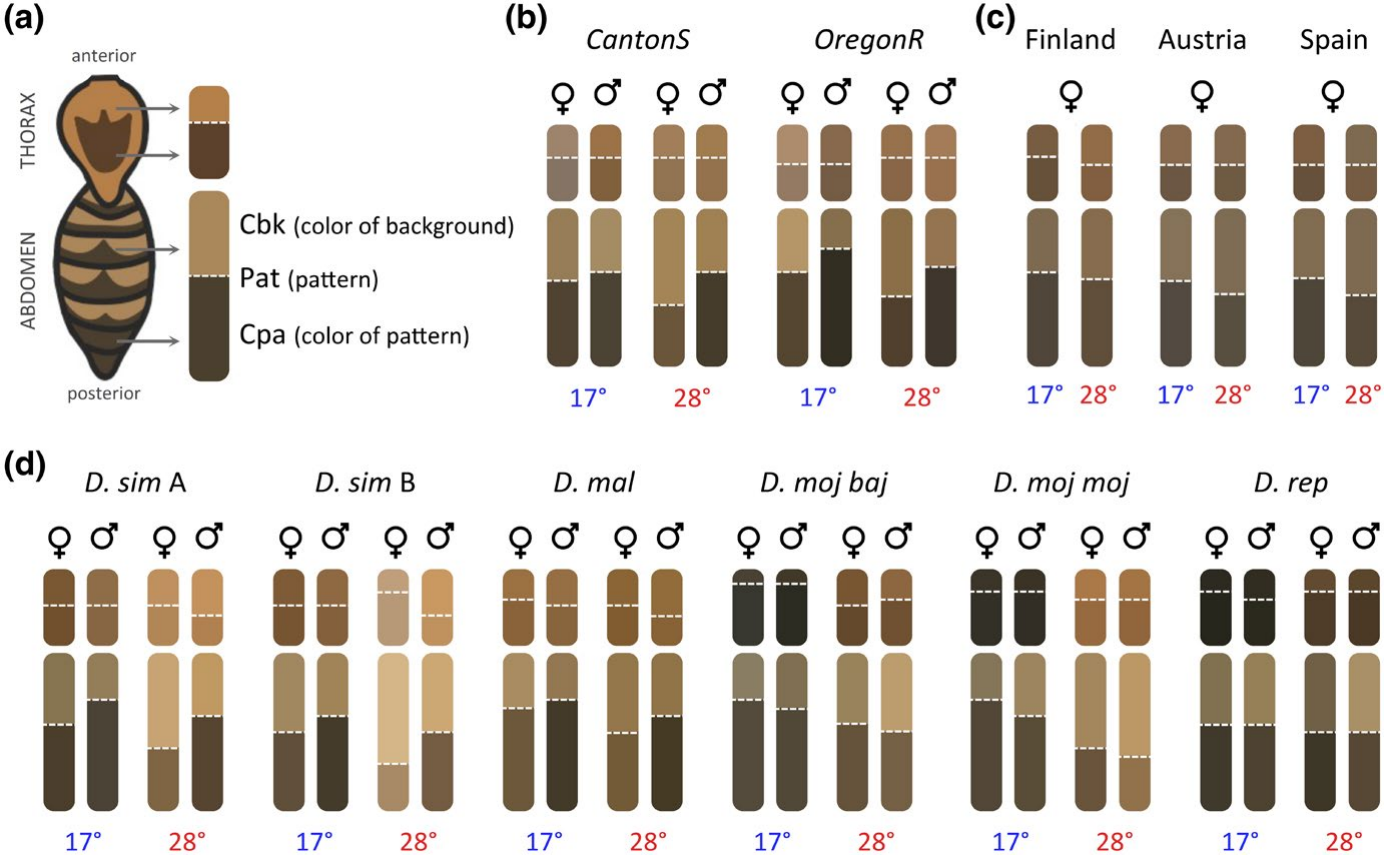
Intra‐ and interspecific variation in *Drosophila* pigmentation. (a) Illustration of a *D. melanogaster* headless body showing the dorsal side of the thorax and abdomen and the scheme we used to represent pigmentation traits for thorax (top rounded rectangle) and abdomen (bottom rounded rectangle). For each of these, the horizontal dashed line separates the color of pattern element (Cpa) and the color of background (Cbk). These are shown in mean color (RGB values) for same‐group individuals, and the height of the dashed line represents the proportion of the transect that is occupied by pattern versus background (Pat). See more details in Figure 1. (b) Pigmentation schemes per strain, sex, and temperature in *D. melanogaster* laboratory populations. (c) Pigmentation schemes in *D. melanogaster* clinal populations, showing mean values from the five genotypes (i.e., isogenic lines) per location. (d) Pigmentation schemes in five *Drosophila* species with one genetic background per species except *D. simulans* where two genetic backgrounds (*D. sim* A and *D. sim* B) were studied

The sequence of averaged RGB pixel values corresponding to each transect was then used to define each of the five pigmentation components as follows. For each pixel, we calculated a normalized darkness value as Dmax–Dbk, where Dmax is the largest possible Euclidean distance between two colors in the RGB color space (in this case Dmax = $\sqrt{3}$), and Dbk is the distance of the pixel's color coordinates to the color black (R = 0, G = 0, B = 0). Overall darkness (Odk) was calculated as the sum of the normalized darkness values for each pixel divided by the number of pixels in the transect. Taking the sequence of normalized darkness values along a transect, we estimated its two enveloping lines (blue and green lines in Figure 1a) by calculating the baselines of the original and negated values using the Statistics‐sensitive Non‐linear Iterative Peak‐clipping (SNIP) algorithm (Ryan et al., 1988). The median line of this envelope (red line in Figure 1a) was then used to separate the transect pixels into two clusters, where the pixels above or below this line correspond, respectively, to the pattern element (trident in the thorax and darker bands in the abdomen) or to the background. Pattern (Pat) was calculated as the proportion of pixels corresponding to the pattern element relative to the transect length. Color of the pattern element (Cpa) is the angle defined in the RGB color space between the best‐fitted line going through the color coordinates of the pixels in the transect that correspond to the pattern element (trident and/or darker bands) in the transect and the gray vector (the black to white diagonal in the RGB color space). Similarly, color of the background (Cbk) was calculated as the angle between the best‐fitted line that goes through the color coordinates of the background pixels in the transect and the gray vector. Pixels corresponding to pattern element and/or background were defined by grouping all RGB values in the transect into two clusters each containing 95% of the light or dark pixels, respectively. Range (Ran) was calculated as the Euclidean distance between the median values of the 20 darkest and the 20 lightest pixels along the transects. The colors represented in Figure 2 correspond to the mean R, mean G, and mean B values for each strain/species, sex, and temperature, which were calculated from Cpa for color of pattern elements and from Cbk for color of the background, respectively.

### 2.3 Statistical analyses

All analyses were conducted in R v 3.6.2 (R Core Team, 2019), using the following R packages: *tidyr* (Wickham & Henry, 2020) to arrange datasets, *ggplot2* (Wickham, 2009) to produce plots, *lme4* (Bates et al., 2015) and *lmerTest* (Kuznetsova et al., 2017) to perform linear mixed‐effects models, *Hmisc* (Harrell et al., 2020) and *corrplot* (Taiyun & Viliam, 2017) to compute correlation matrices, and *emmeans* (Lenth et al., 2018) to perform post hoc pairwise comparisons between groups. The statistical models described below are given in package‐specific R syntax (shown in italics).

Multivariate multiple regression was performed for the data on *D. melanogaster* laboratory populations to test for the effect of strain, sex, temperature (fixed explanatory variables), and interaction terms in all pigmentation traits by combining all traits using the *cbind* function (model *lm(cbind(Odk, Pat, Ran, Cbk, Cpa)* ~ Strain * Sex * Temperature)). A similar analysis was performed for the data on *D. melanogaster* clinal populations testing for the fixed effects and interactions of location, genotype (i.e., isogenic line; nested within location), and temperature (model *lm(cbind(Odk, Pat, Ran, Cbk, Cpa)* ~ Location * Genotype * Temperature), and for the *Drosophila* species, testing for the fixed effects and interactions of species, strain (nested within species), sex, and temperature (model *lm(cbind(Odk, Pat, Ran, Cbk, Cpa)* ~ Species * Species/Strain * Sex * Temperature)), where *Strain* corresponds to the different genetic backgrounds analyzed in *D. melanogaster* (CanS and OreR) and in *D. simulans* (*D. sim* A and *D. sim* B).

Linear mixed effect models were then used to test for the (fixed) effects of different explanatory fixed variables (strains, genotypes or species, sex, and temperature) and their interactions on each of the pigmentation traits (noted as *trait* in the model notations below). *Replicate*, which corresponds to each independent cohort of flies (for any given species/genotype/temperature), was included as random effect in the models (denoted as *(1|Replicate)* in the R syntax below). For *D. melanogaster* laboratory strains: model *lmer(Trait* *~ Sex* * Temperature + (1|Replicate)). The same model was used for all *Drosophila* species, except for *D. simulans*, where we also included the factor *Strain* which corresponds to the different genetic backgrounds studied in this species (*D. sim* A and *D. sim* B) (model: *lmer*(*Trait* *~ Strain* * Sex * Temperature + (1|Replicate))). For the clinal populations: model: *lmer(Trait ~ Location* * Location/Genotype * Temperature + (1|Replicate)). For all the aforementioned mixed models, we used Satterthwaite's method (via *ANOVA* function in *lmerTest* package; Kuznetsova et al., 2017) for approximating degrees of freedom and estimating *F*‐statistics and *p*‐values. For the data on the sensitive stages of development, we used linear effect models to test for the effect and interaction of strain and thermal regime (model: *lm(Trait ~ Strain* * Regime)).

We used *post hoc* pairwise comparisons (Tukey's honest significant differences) to identify differences between strains, sexes, temperatures, and/or thermal regimes. Pearson's correlations were used to check correlations between traits and across temperatures. We used Holm *p*‐value adjustment method to correct for multiple comparisons (Holm, 1979).

## 3 RESULTS

To investigate patterns and sources of variation in *Drosophila* body pigmentation, we quantif​ied five pigmentation traits that include aspects of color and color pattern (see Figure 1 and Materials and Methods). We focused on the dorsal surface of thoraxes and abdomens, characterized for having different types of darker “pattern elements” on a lighter “background” color: a trident at the center of the thorax and bands on each segment of the abdomen. For each body part, we extracted color information along transects running antero‐posteriorly on each body part and quantified a series of pigmentation traits (Figures 1 and 2a): overall darkness (Odk), the relative length of transect occupied by the darker “ornamental” pattern (Pat), the actual color of both background (Cbk) and “ornamental” pattern elements (Cpa), and the distance in RGB space between the darkest and the lightest color that corresponds to the range of color variation (Ran). We investigated how these pigmentation components vary and covary between sexes and between rearing temperatures in *D. melanogaster* representing standard laboratory strains, and natural populations from different geographical locations, as well as in five additional *Drosophila* species. For each dataset (*D. melanogaster* laboratory strains, *D. melanogaster* clinal populations, and *Drosophila* species), the multivariate multiple regression analyses showed that pigmentation differed significantly between strains/genotypes/species, sexes, and temperatures, with effects that depended on body part (Table A1).

### 3.1 Variation in body pigmentation in *D. melanogaster* laboratory populations

**FIGURE 3:**
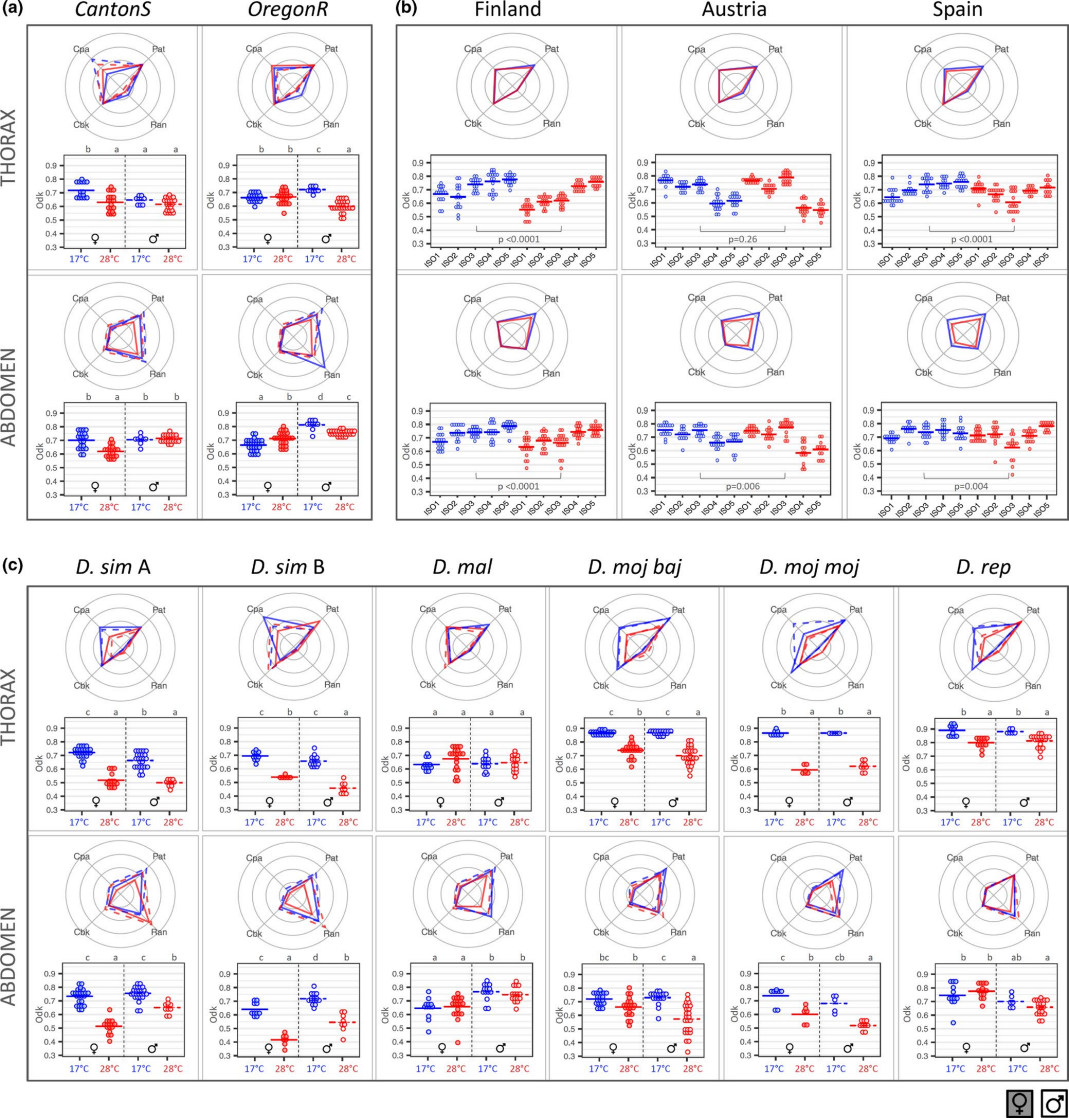
Quantitative phenotyping of *Drosophila* pigmentation component traits. For each population, temperature, sex, and body part, radar plots represent variation for Pat, Ran, Cpa, and Cbk (means; dot plots in Figure S1) and dot plots represent variation for Odk (individual data points and means). Females/males are shown as solid/dashed lines (radar plots) or closed/empty circles (dot plots), and flies reared at 17°C/28°C are shown in blue/red. (a) *D. melanogaster* laboratory populations. Results of statistical test for the effect of sex, temperature, and their interaction on each of the traits are shown in Table A2. Letters in dot plots indicate results of post hoc pairwise comparisons between groups: different letters when significantly different (*p*‐value <.05 for Tukey's honest significance test). (b) *D. melanogaster* clinal populations. For each geographical population, we phenotyped females from five genotypes (i.e., isogenic lines). Results for the effect of location, genotype, and temperature (and interactions) on the different pigmentation traits are in Table A4. Results of the statistical test (*p*‐value) for the effect of temperature on each of the traits are shown in plots. (c) *Drosophila* species. Results of the statistical test for effect of sex, temperature, and their interaction are in Table A5. Letters in dot plots indicate results of post hoc pairwise comparisons between groups: different letters when significantly different (*p*‐value <.05 for Tukey's honest significance test)

**FIGURE 4:**
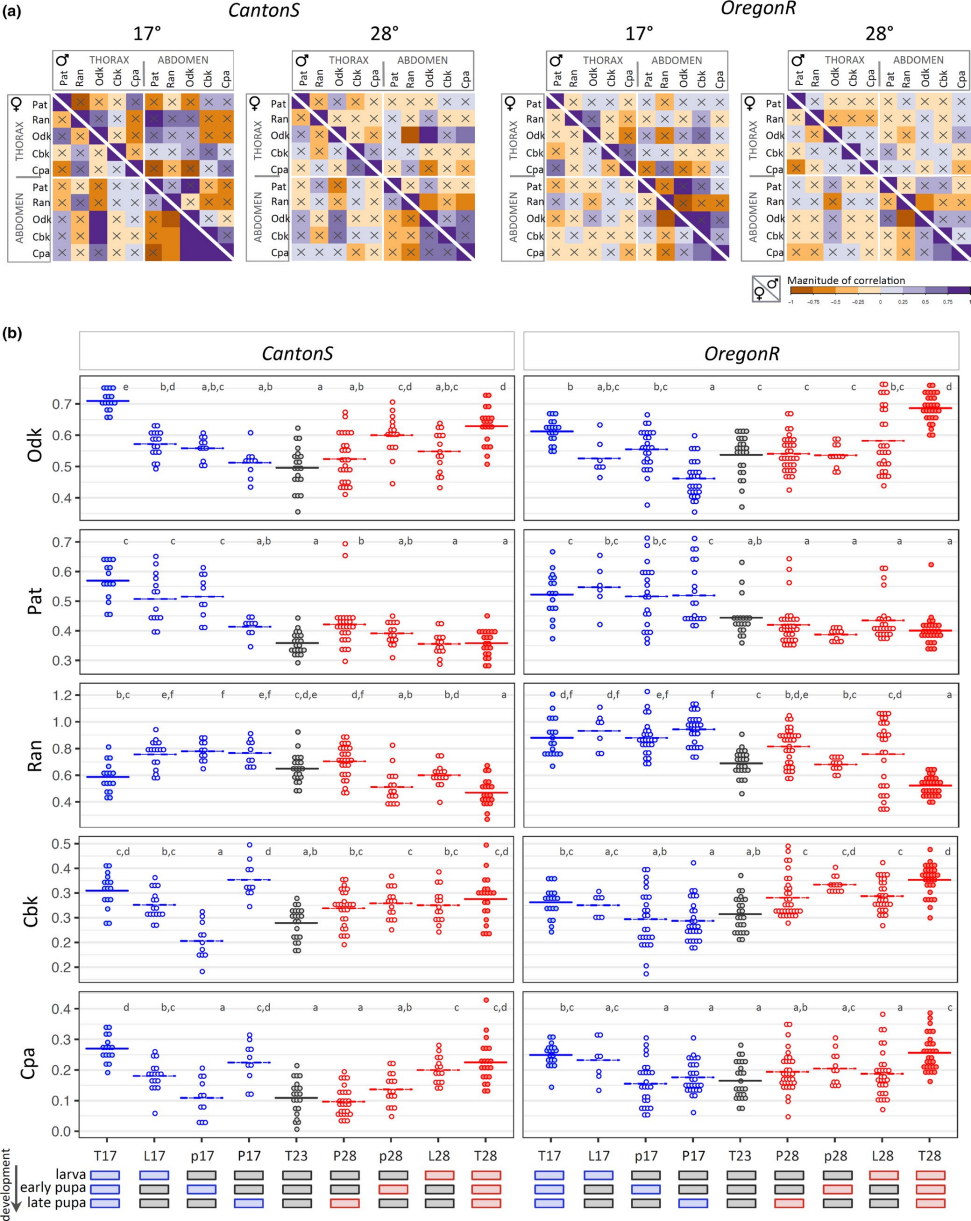
Covariation and thermal sensitivity of *D. melanogaster* pigmentation components. (a) Heat map of Pearson's correlation coefficients for all pigmentation traits in abdomens and thoraxes of CanS (left panels) and OreR (right panels) of flies reared at 17°C or 28°C. For each matrix, females are in the left corner and males in the right. Positive correlations are shown in purple and negative correlations in orange. Correlations not statistically significant (after Holm *p*‐value adjustment method for multiple comparisons) are indicated with a cross. (b) Pigmentation traits (*y*‐axis) in females of two *D. melanogaster* genetic backgrounds (CanS and OreR) exposed to each of the temperature regimes during development (*x*‐axis). The thermal regimes codes and corresponding stages that were exposed to either 17°C or 28°C (instead of the basal temperature of 23°C) were as follows: *T* (constant temperature), *L* (late larval development), *p* (early pupal period), and *P* (late pupal period). In each graph, dots represent phenotypes of single individual females, and the horizontal bar is the mean of those values. The results of the test for differences between strains and thermal regimes on the different plastic traits are shown in Table A3. Letters indicate results of post hoc pairwise comparisons between groups: different letters when significantly different (*p*‐value <.05 for Tukey's honest significance test)

We reared flies from two common laboratory genetic backgrounds of *D. melanogaster*, Oregon R (OreR) and Canton S (CanS), at either 17°C or 28°C to assess thermal plasticity and sexual dimorphism in our pigmentation traits (Figures 2 and 3, Figure S1a, Table A2). We found that flies reared at lower temperature were generally darker than those reared at higher temperature and that males were generally darker than females (Figures 2b and 3a, Figure S1a), as has been previously described in *D. melanogaster* (e.g., Gibert et al., 2009). Yet, we also found differences between strains and body parts in the extent and sometimes the direction of both thermal plasticity and sexual dimorphism for our pigmentation traits (Figures 2b and 3a, Figure S1a, Table A2), as well as for the correlations between them (Figure 4a).

For overall darkness (Odk; dot plots in Figure 3a), flies reared at 17°C were generally darker than those from 28°C, with the exception of CanS males (where differences were not significant in either body part) and OreR females (where abdomens were darker in flies from 28°C). The abdomens were lighter in females relative to males (except for CanS from 17°C), but the thoraxes were lighter in males relative to females (except for CanS from 28°C and OreR from 17°C). We also observed differences between sexes and temperatures for the other pigmentation traits (Pat, Ran, Cbk, and Cpa; radar plots in Figure 3a; dot plots in Figure S1a, Table A2), which depended on body part. Sexual dimorphism and plasticity were lowest for traits reflecting actual color (i.e., Cbk and Cpa) (Figure S1a). While for the thorax, the most striking differences were seen in Ran (for females between temperatures), for the abdomen they were seen for Pat (distinguishing females from 28°C from others) and Ran (extreme for OreR females) (Figure S1a). Variation was only loosely correlated between traits, with few significant correlations, which differed between genetic backgrounds, sexes, and rearing temperatures (Figure 4a). Overall, correlations between traits were weaker across body parts relative to within body parts and in males relative to females.

For those pigmentation traits found to be thermally plastic (i.e., significant differences between individuals reared at different temperatures; cf. Figure S1a, Table A2), we investigated which stages of development were thermally responsive. To do so, we compared phenotypes between individuals (specifically, female abdomens) differing in temperature only for specific developmental time windows (Figure 4b, Table A3). We tested nine thermal regimes (or treatments), including three with constant temperatures (whole development at 17°C, 23°C, or 28°C, T17, T23, and T28 treatments, respectively) and six where most of the development took place at 23°C and only one specific stage (either late larval, early pupal, or late pupal) took place at 17°C or at 28°C. Differences between constant temperatures (T17, T23, and T28 treatments) revealed thermal reaction norms, i.e., the representation of phenotype as a function of temperature (see Schlichting & Pigliucci, 1998), of different shapes for different pigmentation components: T23 phenotype intermediate between T17 and T28 (Ran in OreR; Figure 4b), equal to one of the extreme temperatures (Pat; Figure 4b), or more extreme than both T17 and T28 (Odk; Figure 4b). The period when exposure to a different temperature significantly affected phenotype also differed between traits and genetic backgrounds (Figure 4b), showing that traits and genetic backgrounds differ not only in the extent of thermal plasticity, but also in what developmental stage is thermally responsive.

### 3.2 Body pigmentation differences in *D. melanogaster* natural populations and *Drosophila* species

**FIGURE 5:**
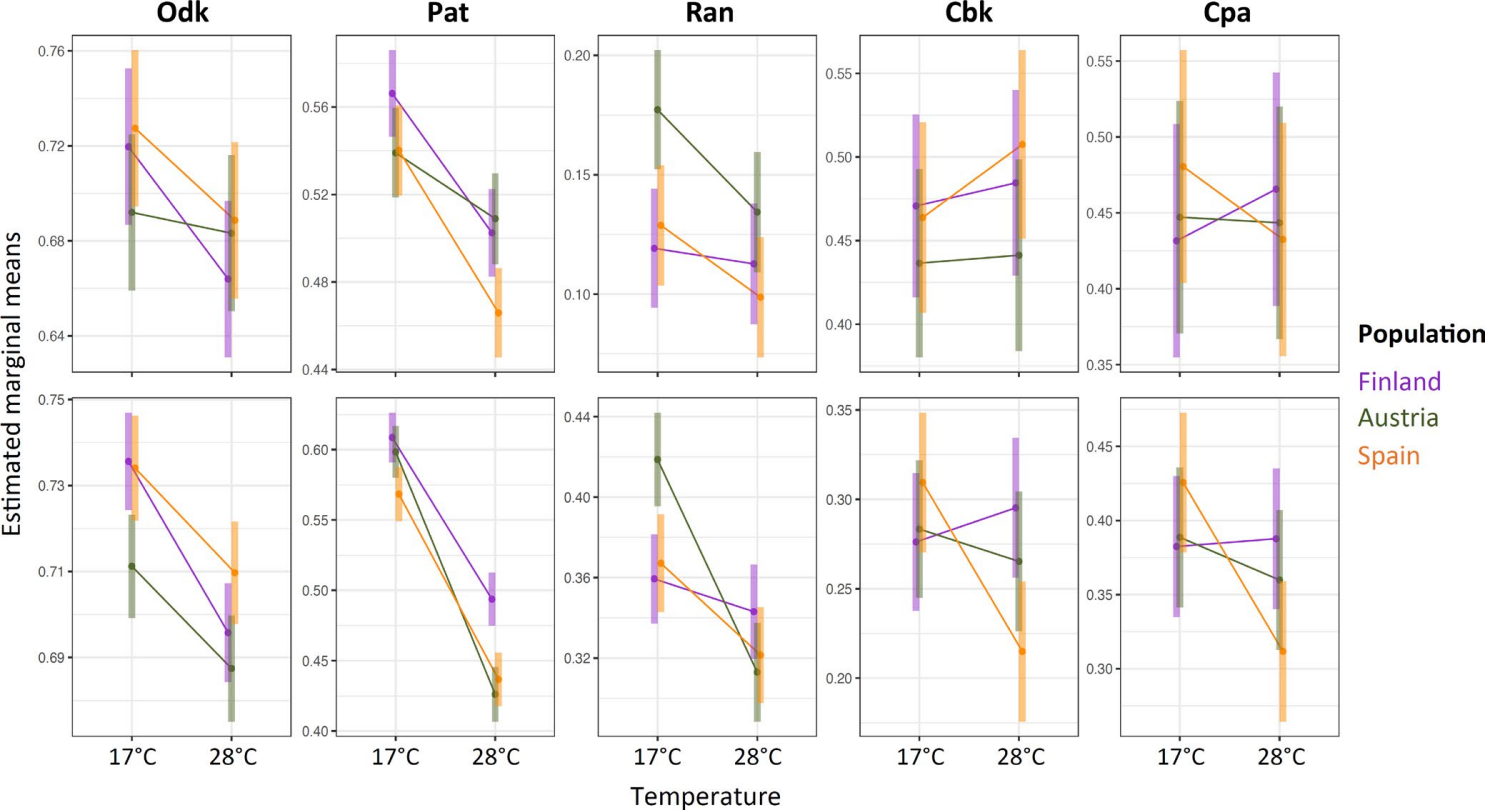
Effects of temperature on pigmentation traits in *D. melanogaster* European populations. Interaction plot showing the estimated marginal means and confidence intervals of all pigmentation traits based on fitted model *lmer(Trait* ~ Location * Location/Genotype * Temperature + (1|Replicate))

We explored patterns of variation in pigmentation components in wild‐caught populations sampled along a latitudinal cline in Europe: Finland, Austria, and Spain (samples from the *DrosEU Consortium;* http://droseu.net/). We quantified pigmentation traits in females from five genotypes (isofemale lines) established from each of the three geographical locations, reared at either 17°C or 28°C. The analysis for each pigmentation component revealed differences between traits in their response to the various explanatory variables and their interactions (Figures 2c and 3b, Table A4). Geographical populations differed in overall darkness (Odk; dot plots in Figure 3b) and in color (both Cbk and Cpa) for the abdomen, but not the thorax (Figure 5, Figure S2, Table A4). Most pigmentation traits (except thorax color; Cpa and Cbk) were thermally plastic, with darker flies for development at 17°C relative to 28°C (Figures 2c and 3b, Figure S2). The northern‐ and southern‐most populations (i.e., Finland and Spain, respectively) did not necessarily show the most extreme phenotypes, neither in terms of overall darkness nor in the extent of plasticity therein (Figure 3b, Figure 5). We also found significant differences between isofemale genotypes (and their plasticity) within each geographical location (Figure 3b, Table A4).

Finally, we assessed pigmentation variation in flies from five additional *Drosophila* species (two genetic backgrounds for *D. simulans* and one genetic background for all other species or subspecies: *D. malerkotliana*, *D*. *repleta*, *D. mojavensis baja*, *D. mojavensis mojavensis*) reared at either 17°C or 28°C (Figures 2d and 3c). We found differences between species in extent and direction of sexual dimorphism and of thermal plasticity for the different pigmentation traits (Figures 2d and 3c, Figure S1b, Table A5). For instance, for Odk (dot plots in Figure 3c), *D. malerkotliana* showed no differences between temperatures and clear differences between sexes, while *D. simulans* had very high thermal plasticity but reduced sexual dimorphism (no differences between females and males reared at 17°C). For the other pigmentation traits (radar plots in Figure 3c and dot plots in Figure S1b), larger differences between sexes and/or temperatures were observed for Pat and/or Ran and less for actual colors (Cpa and Cbk).

## 4 DISCUSSION

We decomposed *Drosophila* body pigmentation into different quantitative traits, including overall darkness (Odk), and traits reflecting properties of color and color pattern (Pat, Ran, Cbk, and Cpa) of both thoraxes and abdomens (Figure 1). We showed differences in trait values, as well as in the extent and direction of thermal plasticity and of sexual dimorphism, for laboratory and natural populations of *D. melanogaster* and several *Drosophila* species (Figures 2, 3, and 5). Different traits, corresponding to different properties of body pigmentation, behaved in a largely independent manner, which was also reflected in low levels of correlations between traits and in differences in the period of development during which traits are thermally responsive (Figure 4).

*Drosophila* pigmentation has been the focus of various studies exploring its ecology, development, and evolution (e.g., Gibert et al., 2017; Kopp et al., 2000; Matute & Harris, 2013; Shearer et al., 2016; Williams et al., 2008). This has provided great insight about the genetic basis and ecological significance of variation, across temporally (e.g., seasonal variation) and geographically (e.g., clinal variation) distinct populations (e.g., Parkash et al., n.d.; Hollocher et al., 2000b; Rajpurohit et al., 2008), as well as across species (Hollocher et al., 2000a, 2000b). Many of those studies focused on specific pigmentation elements in particular species and often used qualitative assessments of pigmentation variation or presence/absence of specific pattern elements (e.g., David et al., 2002; Hollocher et al., 2000). In *D. melanogaster*, for instance, most work has focused on abdominal pigmentation and specifically on the width of the dark bands of the abdominal segments, which is sexually dimorphic (males are generally darker than females; e.g., Kopp et al., 2000) and thermally plastic (flies from lower developmental temperatures are generally darker than flies from higher developmental temperatures; e.g., David et al., 1990; Gibert et al., 2007, 2009). Our analysis, quantifying different properties, including actual color, of both abdomen and thorax pigmentation in *D. melanogaster* and other *Drosophila* species, revealed a more complex picture of variation in body pigmentation. We did not, for example, always find that males were darker than females or that flies reared at lower temperatures were darker than those from higher temperatures. Rather, we found trait specificities in how pigmentation varied between sexes and between developmental temperatures. This was true not only for overall darkness (Odk) of the abdomen, the trait that should be more similar to previous characterizations of abdominal pigmentation (e.g., David et al., 1990; Hollocher et al., 2000a), but also for other properties of body pigmentation, including actual color of background and pattern elements (i.e., abdominal bands and thoracic trident), which had not been investigated before. Moreover, we also showed that pigmentation components, as well as sexual dimorphism and thermal plasticity therein, vary greatly between species, genotypes, and body parts. The mechanisms underlying such intra‐ and interspecific variation in different traits, as well as the trait‐specific responses to temperature, remain to be explored and might involve differences in the environmental sensitivities of the regulatory regions (e.g., enhancers) controlling pigmentation‐related genes (e.g., De Castro et al., 2018).

Our results also show only weak correlations between traits, which differ between sexes and with rearing temperatures (Figure 4a). Environmental effects on trait associations have been described previously; for instance, cold temperature triggered a shift in the sign of the correlation between body size and longevity in *D. melanogaster* (Norry & Loeschcke, 2002). Differing correlations between body parts (or regions within a body part) have also been identified for *D. melanogaster* pigmentation (e.g., Bastide et al., 2014; Gibert et al., 2000), with the extent of genetic correlations decreasing with increasing distance between body segments (Gibert et al., 2000). Ultimately, the dependency of trait associations on genetic and environmental factors has the potential to influence adaptation (e.g., Manenti et al., 2016; Marquez & Knowles, 2007), as evolutionary change can result from both direct and correlated responses to selection (e.g., Rajpurohit & Gibbs, 2012). Altogether, our results suggest a large degree of developmental and evolutionary independence between pigmentation components, which could facilitate the diversification of body coloration in *Drosophila*. This is also apparent in that we find differences between traits in the extent of thermal plasticity and sexual dimorphism (Figures 2 and 3), as well as in which period during development temperature affects adult phenotype (Figure 4b).

Studies exploring the ecological conditions driving the evolution of melanism in *Drosophila* have documented correlations between body pigmentation and several eco‐geographic variables (e.g., latitude, altitude, temperature, humidity) (e.g., Gibert et al., 2016; Rajpurohit et al., 2008; Shearer et al., 2016). Clinal variation in pigmentation has been shown for thoracic trident (e.g., David et al., 1985; Telonis‐Scott et al., 2011) and for abdominal pigmentation (e.g., Das, 2009; Pool & Aquadro, 2007). Generally, darker phenotypes in colder environments (e.g., at high latitudes or altitudes) have been hypothesized to allow flies to better absorb solar radiation (c.f. thermal budget or thermal melanism hypothesis; Clusella‐Trullas et al., 2008; Trullas et al., 2007), to increase desiccation resistance (e.g., Parkash et al., 2008), and/or to provide protection against UV radiation (e.g., Bastide et al., 2014). Plasticity, on the other hand, is expected to be greater in environments that are more variable (Lande, 2014), such as those with larger seasonal fluctuations, often occurring at higher latitudes. However, our analysis of the pigmentation patterns from *D. melanogaster* populations collected along a European latitude cline (Finland, Austria, Spain) did not always revealed darker pigmentation nor higher plasticity in the northern‐most population (Finland). This may reflect that other environmental parameters and ecological conditions—not considered in our study—could account for the differences between populations in the pigmentation components. It is also possible that having only three populations from three latitudes may be limiting our assessment of latitudinal patterns in pigmentation and plasticity therein.

In terms of a function in thermoregulation favoring darker flies in cooler environments (David et al., 1985; Hollocher et al., 2000a; Matute & Harris, 2013; Shearer et al., 2016; Wittkopp et al., 2011), we could expect our trait overall darkness (Odk) to be the most relevant trait. Our analyses revealed that flies can become overall darker (higher Odk) by changing actual colors of background or of ornamental elements (Cbk and Cpa, respectively) or the proportion of the abdomen/thorax length covered with the darker bands/trident (Pat). For instance, males of CanS reared at 17°C and 28°C show the same overall darkness (Odk), but differ in what pigmentation components make that up; Odk is mostly determined by color components at 17°C and by color pattern components at 28°C (i.e., Cpa and Cbk are lower, while Pat and Ran are higher at 17°C than at 28°C). It is unclear whether these traits are mere components of the overall body darkness (Odk) or can themselves be under direct natural selection.

Variation in pigmentation between body parts, individuals, populations, and species can be caused by differences in actual color and/or in how colors are spatially organized to make up color patterns (Nijhout, 2010; Wittkopp & Beldade, 2009). However, seldom do studies of animal pigmentation consider and quantify distinct pigmentation component traits, and the extent to which they might be differently affected by genetic and/or environmental factors, and might develop and evolve more or less independently from each other. The increased attention to studying the mechanisms underlying phenotypic variation resulted in great detail and sophistication in the characterization of its genetic underpinnings. However, the detail in describing and quantifying phenotypes has lagged behind. The lack of quantitative methods for phenotyping (see Gerlai, 2002; Houle et al., 2010) can result in an oversimplification of complex phenotypes, dismissing that those phenotypes are often made up of distinct component traits that can respond to internal and external factors in different manners (e.g., Mateus et al., 2014; Vrieling et al., 1994). We attempted to provide a better resolution of variation in *Drosophila* body color, a visually compelling example of adaptive evolution. Combining it with existing genetic resources and with access to natural variation can provide a deeper resolution of the patterns and processes underlying phenotypic variation, within and between species.

## CONFLICT OF INTEREST

The authors declare that no conflict of interest exists. The funders had no role in study design, data collection and analysis, decision to publish, or preparation of the manuscript.

## AUTHOR CONTRIBUTIONS

**Elvira Lafuente:** Conceptualization (equal); data curation (lead); formal analysis (lead); funding acquisition (supporting); investigation (lead); methodology (equal); project administration (equal); supervision (supporting); visualization (equal); writing‐original draft (lead); writing‐review & editing (lead). **Filipa Alves:** Formal analysis (equal); methodology (equal); software (equal); visualization (supporting); writing‐review & editing (supporting). **Jessica G. King:** Formal analysis (supporting); investigation (equal); writing‐review & editing (supporting). **Carolina M. Peralta:** Investigation (equal); visualization (equal); writing‐review & editing (supporting). **Patrícia Beldade:** Conceptualization (equal); formal analysis (supporting); funding acquisition (lead); methodology (equal); project administration (lead); resources (lead); supervision (lead); visualization (equal); writing‐original draft (supporting); writing‐review & editing (equal).

## Supporting information

Fig S1‐2

## Data Availability

Data are available from Dryad Data Repository at https://doi.org/10.5061/dryad.kwh70rz3m.

## APPENDIX A

**TABLE A1:** Pigmentation in *D. melanogaster* populations and *Drosophila* species

		THORAX						ABDOMEN					
		*Df*	Pillai	approx *F*	num *Df*	den *Df*	Pr(>*F*)	*Df*	Pillai	approx *F*	num *Df*	den *Df*	Pr(>*F*)
*D. melanogaster* laboratory populations	(Intercept)	1	1.00	7,342.50	5	132	<2.2E−16	1	1.00	41,547.00	5	153	<2.2E−16
	Strain	1	0.04	1.10	5	132	3.63E−01	1	0.58	42.00	5	153	<2.2E−16
	Temperature	1	0.65	49.70	5	132	<2.2E−16	1	0.69	69.00	5	153	<2.2E−16
	Sex	1	0.33	12.70	5	132	4.11E−07	1	0.84	162.00	5	153	<2.2E−16
	Strain:Temperature	1	0.03	0.80	5	132	5.42E−01	1	0.22	9.00	5	153	2.20E−04
	Strain:Sex	1	0.05	1.40	5	132	2.17E−01	1	0.38	19.00	5	153	1.48E−11
	Temperature:Sex	1	0.08	2.20	5	132	6.19E−02	1	0.55	38.00	5	153	<2.2E−16
	Strain:Temperature:Sex	1	0.20	6.70	5	132	1.24E−02	1	0.26	11.00	5	153	7.47E−06
	Residuals	136						157					
*D. melanogaster* European populations	(Intercept)	1	1.00	26,858.70	5	456	<2.2E−16	1	1.00	59,146	5	464	<2.2E−16
	Location	2	0.22	11.10	10	914	<2.2E−16	2	0.24	13	10	930	<2.2E−16
	Temperature	1	0.28	35.10	5	456	<2.2E−16	1	0.68	193	5	464	<2.2E−16
	Location:Genotype	12	1.15	11.40	60	2,300	<2.2E−16	12	0.96	9	60	2,340	<2.2E−16
	Location:Temperature	2	0.11	5.50	10	914	4.99E−08	2	0.30	17	10	930	<2.2E−16
	Location:Genotype:Temperature	12	0.45	3.80	60	2,300	<2.2E−16	12	0.42	4	60	2,340	<2.2E−16
	Residuals	460						468					
*Drosophila* species	(Intercept)	1	1.00	13,874.30	5	238	<2.2E−16	1	1.00	49,795.00	5	261	<2.2E−16
	Species	1	0.48	43.60	5	238	<2.2E−16	1	0.36	30.00	5	261	<2.2E−16
	Temperature	1	0.70	108.50	5	238	<2.2E−16	1	0.75	156.00	5	261	<2.2E−16
	Sex	1	0.29	19.10	5	238	5.30E−13	1	0.83	254.00	5	261	<2.2E−16
	Species:Strain	2	0.07	1.80	10	478	5.51E−02	2	0.60	23.00	10	524	<2.2E−16
	Species:Temperature	1	0.49	46.30	5	238	2.20E−16	1	0.48	49.00	5	261	<2.2E−16
	Species:Sex	1	0.15	8.40	5	238	2.71E−04	1	0.11	7.00	5	261	5.99E−06
	Temperature:Sex	1	0.03	1.50	5	238	1.85E−01	1	0.47	46.00	5	261	<2.2E−16
	Species:Strain:Temperature	2	0.07	1.90	10	478	5.00E−02	2	0.47	16.00	10	524	<2.2E−16
	Species:Strain:Sex	2	0.08	2.00	10	478	2.99E−02	2	0.37	12.00	10	524	<2.2E−16
	Species:Temperature:Sex	1	0.05	2.60	5	238	2.76E−02	1	0.14	8.00	5	261	3.08E−07
	Species:Strain:Temperature:Sex	2	0.21	5.50	10	478	1.03E−04	2	0.24	7.00	10	524	1.46E−10
	Residuals	242						265					

Note

Results of analysis of variance for multivariate multiple regressions performed on each dataset, for the effects of different factors on pigmentation of each body part. Non‐significant effects (*p*‐value >.05) are shown in grey. For *D. melanogaster* laboratory populations, table shows the results for the effect and interactions of strain, sex, and temperature (model: *lm(cbind(Pat, Ran, Odk, Cbk, Cpa)* *~ Strain * Sex * Temperature)*) on pigmentation of each body part. For *D. melanogaster* European populations, table shows the results for the effect and interactions of location, genotype (i.e. isogenic line), and temperature (model *lm(cbind(Odk, Pat, Ran, Cbk, Cpa)* *~ Location * Genotype * Temperature)*). For *Drosophila* species, table shows the results for the effect and interactions of species, strain (nested within species), sex, and temperature (model *lm(cbind(Odk, Pat, Ran, Cbk, Cpa)* *~ Species * Species/Strain * Sex * Temperature))*. The factor *Strain* corresponds to the different genetic backgrounds analyzed in *D. melanogaster* (CanS and OreR) and in *D. simulans* (*D.sim* A and *D.sim* B).

**TABLE A2:** Pigmentation components in *D. melanogaster* laboratory populations

			THORAX						THORAX					
			Sum Sq	Mean Sq	NumDF	DenDF	*F* value	Pr(>*F*)	Sum Sq	Mean Sq	NumDF	DenDF	*F* value	Pr(>*F*)
CantonS	Pat	Sex	0.00	0.00	1	60.98	0.08	7.84E−01	0.29	0.29	1	69.49	186.01	<2.2E−16
		Temperature	0.01	0.01	1	60.84	0.99	3.24E−01	0.16	0.16	1	69.02	101.82	3.20E−15
		Sex:Temperature	0.00	0.00	1	57.63	0.41	5.27E−01	0.02	0.02	1	69.93	14.98	2.42E−04
	Odk	Sex	0.01	0.01	1	59.75	12.42	8.23E−04	0.04	0.04	1	69.58	34.76	1.21E−07
		Temperature	0.10	0.10	1	59.45	92.03	1.10E−13	0.04	0.04	1	69.20	28.86	9.87E−07
		Sex:Temperature	0.00	0.00	1	60.26	3.30	7.43E−02	0.01	0.01	1	69.94	10.51	1.82E−03
	Ran	Sex	0.04	0.04	1	58.01	18.82	5.81E−05	0.22	0.22	1	69.98	29.03	9.08E−07
		Temperature	0.09	0.09	1	52.30	40.07	5.65E−08	0.06	0.06	1	69.75	8.38	5.06E−03
		Sex:Temperature	0.00	0.00	1	26.61	0.05	8.30E−01	0.04	0.04	1	68.07	5.43	2.28E−02
	Cbk	Sex	0.01	0.01	1	61.00	0.63	4.31E−01	0.05	0.05	1	69.97	23.04	8.71E−06
		Temperature	0.01	0.01	1	61.00	0.45	5.04E−01	0.00	0.00	1	69.72	0.68	4.11E−01
		Sex:Temperature	0.03	0.03	1	61.00	2.06	1.56E−01	0.01	0.01	1	68.22	4.32	4.14E−02
	Cpa	Sex	1.09	1.09	1	59.93	6.89	1.10E−02	0.00	0.00	1	69.95	0.43	5.14E−01
		Temperature	0.00	0.00	1	59.98	0.02	8.97E−01	0.01	0.01	1	69.68	2.48	1.19E−01
		Sex:Temperature	0.06	0.06	1	45.53	0.38	5.39E−01	0.02	0.02	1	68.57	5.46	2.24E−02
OregonR	Pat	Sex	0.00	0.00	1	75.00	0.03	8.72E−01	0.49	0.49	1	32.94	212.38	6.42E−16
		Temperature	0.05	0.05	1	75.00	2.10	1.52E−01	0.31	0.31	1	6.53	134.38	1.36E−05
		Sex:Temperature	0.00	0.00	1	75.00	0.03	8.60E−01	0.00	0.00	1	8.05	0.11	7.51E−01
	Odk	Sex	0.00	0.00	1	37.60	0.00	9.62E−01	0.16	0.16	1	34.41	103.39	6.64E−12
		Temperature	0.05	0.05	1	48.99	33.71	4.66E−07	0.00	0.00	1	12.27	0.15	7.02E−01
		Sex:Temperature	0.04	0.04	1	20.33	25.91	5.33E−05	0.05	0.05	1	10.78	31.26	1.75E−04
	Ran	Sex	0.06	0.06	1	75.00	25.53	2.98E−06	0.22	0.22	1	37.58	23.12	2.47E−05
		Temperature	0.13	0.13	1	75.00	53.46	2.41E−10	0.30	0.30	1	26.53	31.86	5.76E−06
		Sex:Temperature	0.03	0.03	1	75.00	11.88	9.33E−04	0.54	0.54	1	15.57	56.50	1.46E−06
	Cbk	Sex	0.06	0.06	1	75.00	0.77	3.84E−01	0.02	0.02	1	42.20	20.72	4.47E−05
		Temperature	0.00	0.00	1	75.00	0.03	8.73E−01	0.01	0.01	1	28.86	12.01	1.68E−03
		Sex:Temperature	0.03	0.03	1	75.00	0.43	5.14E−01	0.00	0.00	1	18.09	0.07	7.98E−01
	Cpa	Sex	0.01	0.01	1	45.60	0.06	8.02E−01	0.05	0.05	1	44.08	17.31	1.45E−04
		Temperature	0.00	0.00	1	66.43	0.00	9.85E−01	0.01	0.01	1	25.02	3.87	6.02E−02
		Sex:Temperature	0.02	0.02	1	29.58	0.13	7.24E−01	0.00	0.00	1	17.97	0.31	5.85E−01

Note

Results of analysis of variance for the effect and interaction of sex and temperature on the different pigmentation traits per body part (model: *lm(Trait* *~ Sex * Temperature + (1|Replicate)*). Nonsignificant effects (*p*‐value >.05) are shown in grey.

**TABLE A3:** Windows of sensitivity for pigmentation plasticity in *D. melanogaster*

		*Df*	Sum Sq	Mean Sq	*F* value	Pr(>*F*)
Pat	Strain	1	0.08	0.08	17.32	4.09E−05
	Regime	8	1.07	0.13	28.65	<2.2e−16
	Strain:Regime	8	0.17	0.02	4.42	4.40E−05
	Residuals	312	1.46	0.00		
Odk	Strain	1	0.00	0.00	1.02	3.13E−01
	Regime	8	1.25	0.16	40.31	<2.2e−16
	Strain:Regime	8	0.22	0.03	6.96	1.63E−08
	Residuals	347	1.35	0.00		
Ran	Strain	1	1.73	1.73	101.40	<2.2e−16
	Regime	8	4.94	0.62	36.20	<2.2e−16
	Strain:Regime	8	0.45	0.06	3.33	1.11E−03
	Residuals	347	5.92	0.02		
Cbk	Strain	1	0.01	0.01	3.93	4.82E−02
	Regime	8	0.20	0.02	17.24	<2.2e−16
	Strain:Regime	8	0.10	0.01	8.98	3.32E−11
	Residuals	347	0.49	0.00		
Cpa	Strain	1	0.09	0.09	26.16	5.22E−07
	Regime	8	0.63	0.08	22.36	<2.2e−16
	Strain:Regime	8	0.17	0.02	6.15	2.00E−07
	Residuals	347	1.23	0.00		

Note

Results of analysis of variance for the effect and interaction of strain and thermal regime on the different pigmentation traits per body part (model: *lm(Trait* *~ Strain*Regime)*).

**TABLE A4:** Pigmentation components in *D. melanogaster* European populations

		THORAX						ABDOMEN					
		Sum Sq	Mean Sq	NumDF	DenDF	*F* value	Pr(>*F*)	Sum Sq	Mean Sq	NumDF	DenDF	*F* value	Pr(>*F*)
Pat	Location	0.26	0.13	2	460.00	15.56	2.88E−07	0.00	0.00	2	467.79	0.51	6.02E−01
	Temperature	0.38	0.38	1	460.00	46.39	3.05E−11	2.34	2.34	1	428.39	655.94	<2.2E−16
	Location:Genotype	1.13	0.09	12	460.00	11.42	<2.2E−16	0.81	0.07	12	465.92	18.88	<2.2E−16
	Location:Temperature	0.08	0.04	2	460.00	5.01	7.05E−03	0.03	0.02	2	467.79	4.34	1.36E−02
	Location:Genotype:Temperature	0.33	0.03	12	460.00	3.34	1.14E−04	0.16	0.01	12	465.78	3.82	1.39E−05
Odk	Location	0.39	0.19	2	458.59	83.57	<2.2E−16	0.18	0.09	2	468.00	31.62	1.32E−13
	Temperature	0.13	0.13	1	457.75	58.55	1.18E−13	0.11	0.11	1	468.00	37.18	2.26E−09
	Location:Genotype	1.72	0.14	12	457.36	62.25	<2.2E−16	0.96	0.08	12	468.00	28.20	<2.2E−16
	Location:Temperature	0.15	0.07	2	458.59	31.66	1.32E−13	0.02	0.01	2	468.00	2.84	5.96E−02
	Location:Genotype:Temperature	0.33	0.03	12	457.35	11.82	<2.2E−16	0.22	0.02	12	468.00	6.53	8.16E−11
Ran	Location	0.20	0.10	2	458.86	52.31	<2.2E−16	0.57	0.29	2	464.52	35.87	3.25E−15
	Temperature	0.08	0.08	1	453.44	42.78	1.66E−10	0.38	0.38	1	359.23	47.39	2.61E−11
	Location:Genotype	0.27	0.02	12	457.32	12.14	<2.2E−16	2.46	0.20	12	459.50	25.61	<2.2E−16
	Location:Temperature	0.03	0.02	2	458.86	8.77	1.83E−04	0.06	0.03	2	464.52	4.02	1.85E−02
	Location:Genotype:Temperature	0.05	0.00	12	457.30	2.31	7.10E−03	0.62	0.05	12	459.60	6.45	1.21E−10
Cbk	Location	0.20	0.10	2	459.30	1.63	1.97E−01	0.11	0.05	2	468.18	10.05	5.32E−05
	Temperature	0.05	0.05	1	452.16	0.85	3.58E−01	0.11	0.11	1	460.04	20.82	6.47E−06
	Location:Genotype	2.00	0.17	12	458.86	2.70	1.54E−03	0.83	0.07	12	466.53	12.64	<2.2E−16
	Location:Temperature	0.05	0.03	2	459.36	0.44	6.47E−01	0.04	0.02	2	468.18	3.95	2.00E−02
	Location:Genotype:Temperature	2.16	0.18	12	459.17	2.93	6.18E−04	0.26	0.02	12	466.45	4.03	5.61E−06
Cpa	Location	0.12	0.06	2	458.80	4.78	8.78E−03	0.05	0.02	2	467.51	6.20	2.20E−03
	Temperature	0.00	0.00	1	458.04	0.31	5.76E−01	0.24	0.24	1	467.92	60.79	4.16E−14
	Location:Genotype	1.29	0.11	12	457.74	8.56	9.71E−15	0.43	0.04	12	466.37	8.89	2.08E−15
	Location:Temperature	0.02	0.01	2	458.80	0.66	5.19E−01	0.03	0.01	2	467.51	3.18	4.26E−02
	Location:Genotype:Temperature	0.45	0.04	12	457.73	2.98	5.03E−04	0.18	0.01	12	466.33	3.74	1.99E−05

Note

Results of analysis of variance for the effect and interaction of location, genotype (i.e. isogenic line) and temperature on the different pigmentation traits per body part (model: *lm(Trait* *~ Location *Location/Genotype * Temperature + (1|Replicate)*). Non‐significant effects (*p*‐value >.05) are shown in grey.

**TABLE A5:** Pigmentation components in *Drosophila* species

			THORAX						ABDOMEN					
			Sum Sq	Mean Sq	NumDF	DenDF	*F* value	Pr(>*F*)	Sum Sq	Mean Sq	NumDF	DenDF	*F* value	Pr(>*F*)
*D.sim*	Pat	Strain	0.02	0.02	1	106.00	1.46	2.30E−01	0.13	0.13	1	64.28	62.86	4.19E−11
		Sex	0.29	0.29	1	106.00	19.97	1.98E−05	0.54	0.54	1	108.00	272.49	<2.20E−16
		Temperature	0.01	0.01	1	106.00	0.76	3.84E−01	0.50	0.50	1	106.54	252.49	<2.20E−16
		Strain:Sex	0.03	0.03	1	106.00	1.82	1.80E−01	0.00	0.00	1	108.00	0.55	4.59E−01
		Strain:Temperature	0.07	0.07	1	106.00	4.77	3.11E−02	0.00	0.00	1	106.54	1.86	1.76E−01
		Sex:Temperature	0.05	0.05	1	106.00	3.30	7.21E−02	0.03	0.03	1	107.19	15.94	1.20E−04
		Strain:Sex:Temperature	0.03	0.03	1	106.00	2.38	1.26E−01	0.00	0.00	1	107.19	0.45	5.03E−01
	Odk	Strain	0.01	0.01	1	105.92	3.79	5.43E−02	0.07	0.07	1	103.46	29.29	4.05E−07
		Sex	0.08	0.08	1	104.47	54.68	3.68E−11	0.16	0.16	1	107.27	68.01	4.44E−13
		Temperature	0.61	0.61	1	104.84	437.62	<2.20E−16	0.65	0.65	1	107.89	267.48	<2.20E−16
		Strain:Sex	0.00	0.00	1	104.47	0.00	9.84E−01	0.01	0.01	1	107.27	3.07	8.28E−02
		Strain:Temperature	0.00	0.00	1	104.84	0.56	4.55E−01	0.02	0.02	1	107.89	6.40	1.29E−02
		Sex:Temperature	0.00	0.00	1	104.16	0.25	6.15E−01	0.04	0.04	1	106.58	16.21	1.06E−04
		Strain:Sex:Temperature	0.01	0.01	1	104.16	5.06	2.66E−02	0.01	0.01	1	106.58	2.37	1.26E−01
	Ran	Strain	0.00	0.00	1	33.51	4.52	4.10E−02	0.01	0.01	1	98.51	0.77	3.82E−01
		Sex	0.00	0.00	1	105.17	1.33	2.51E−01	0.37	0.37	1	107.52	38.17	1.18E−08
		Temperature	0.03	0.03	1	101.69	40.40	5.95E−09	0.23	0.23	1	108.00	22.95	5.33E−06
		Strain:Sex	0.00	0.00	1	105.17	5.69	1.89E−02	0.07	0.07	1	107.52	6.71	1.09E−02
		Strain:Temperature	0.01	0.01	1	101.69	10.98	1.28E−03	0.27	0.27	1	108.00	27.22	8.83E−07
		Sex:Temperature	0.00	0.00	1	105.47	0.47	4.96E−01	0.17	0.17	1	106.74	17.79	5.19E−05
		Strain:Sex:Temperature	0.01	0.01	1	105.47	17.51	5.93E−05	0.13	0.13	1	106.74	13.13	4.48E−04
	Cbk	Strain	0.29	0.29	1	106.00	5.41	2.20E−02	0.01	0.01	1	107.92	7.14	8.70E−03
		Sex	0.09	0.09	1	106.00	1.74	1.90E−01	0.17	0.17	1	106.47	125.72	<2.20E−16
		Temperature	0.02	0.02	1	106.00	0.29	5.93E−01	0.00	0.00	1	106.89	1.12	2.92E−01
		Strain:Sex	0.58	0.58	1	106.00	10.93	1.29E−03	0.00	0.00	1	106.47	2.77	9.89E−02
		Strain:Temperature	0.28	0.28	1	106.00	5.23	2.42E−02	0.05	0.05	1	106.89	34.93	4.13E−08
		Sex:Temperature	0.13	0.13	1	106.00	2.41	1.23E−01	0.03	0.03	1	106.19	25.69	1.71E−06
		Strain:Sex:Temperature	0.04	0.04	1	106.00	0.77	3.81E−01	0.00	0.00	1	106.19	3.54	6.27E−02
	Cpa	Strain	0.33	0.33	1	77.57	3.50	6.51E−02	0.10	0.10	1	41.27	34.47	6.45E−07
		Sex	0.01	0.01	1	105.92	0.13	7.16E−01	0.16	0.16	1	107.59	55.86	2.19E−11
		Temperature	1.16	1.16	1	105.72	12.21	6.94E−04	0.07	0.07	1	103.58	24.11	3.41E−06
		Strain:Sex	0.00	0.00	1	105.92	0.00	9.65E−01	0.03	0.03	1	107.59	8.96	3.42E−03
		Strain:Temperature	0.14	0.14	1	105.72	1.42	2.35E−01	0.00	0.00	1	103.58	0.06	8.13E−01
		Sex:Temperature	0.30	0.30	1	104.83	3.18	7.74E−02	0.00	0.00	1	107.52	0.86	3.55E−01
		Strain:Sex:Temperature	0.26	0.26	1	104.83	2.76	9.99E−02	0.01	0.01	1	107.52	2.03	1.57E−01
*D.mal*	Pat	Sex	0.08	0.08	1	56.99	6.95	1.08E−02	0.13	0.13	1	57.00	24.09	8.08E−06
		Temperature	0.23	0.23	1	54.08	20.61	3.18E−05	0.25	0.25	1	57.00	45.48	8.49E−09
		Sex:Temperature	0.00	0.00	1	56.60	0.23	6.35E−01	0.00	0.00	1	57.00	0.90	3.46E−01
	Odk	Sex	0.00	0.00	1	53.39	0.12	7.34E−01	0.17	0.17	1	56.86	34.80	2.12E−07
		Temperature	0.02	0.02	1	53.88	5.58	2.19E−02	0.00	0.00	1	55.94	0.00	9.90E−01
		Sex:Temperature	0.01	0.01	1	53.34	4.60	3.65E−02	0.01	0.01	1	56.52	1.11	2.96E−01
	Ran	Sex	0.00	0.00	1	57.00	2.12	1.50E−01	0.04	0.04	1	55.94	4.28	4.31E−02
		Temperature	0.02	0.02	1	55.04	10.77	1.80E−03	0.31	0.31	1	56.62	30.35	9.18E−07
		Sex:Temperature	0.00	0.00	1	56.84	0.22	6.38E−01	0.28	0.28	1	55.33	27.49	2.56E−06
	Cbk	Sex	0.03	0.03	1	57.00	0.64	4.26E−01	0.03	0.03	1	55.75	26.06	4.14E−06
		Temperature	0.14	0.14	1	57.00	2.92	9.28E−02	0.00	0.00	1	56.79	1.27	2.65E−01
		Sex:Temperature	0.05	0.05	1	57.00	1.02	3.16E−01	0.00	0.00	1	55.58	1.26	2.66E−01
	Cpa	Sex	0.00	0.00	1	57.00	0.01	9.21E−01	0.08	0.08	1	56.26	13.16	6.18E−04
		Temperature	0.02	0.02	1	57.00	0.35	5.56E−01	0.00	0.00	1	56.91	0.00	9.69E−01
		Sex:Temperature	0.00	0.00	1	57.00	0.08	7.74E−01	0.00	0.00	1	55.93	0.40	5.28E−01
*D.mojbaj*	Pat	Sex	0.03	0.03	1	82.00	4.57	3.56E−02	0.07	0.07	1	79.95	31.44	2.84E−07
		Temperature	0.97	0.97	1	82.00	129.47	<2.00E−16	0.39	0.39	1	66.09	183.19	<2.20E−16
		Sex:Temperature	0.02	0.02	1	82.00	2.20	1.42E−01	0.00	0.00	1	79.99	0.17	6.80E−01
	Odk	Sex	0.01	0.01	1	82.00	3.93	5.07E−02	0.04	0.04	1	79.96	7.30	8.41E−03
		Temperature	0.47	0.47	1	82.00	226.13	<2.00E−16	0.25	0.25	1	71.04	47.44	1.88E−09
		Sex:Temperature	0.01	0.01	1	82.00	4.22	4.31E−02	0.05	0.05	1	79.81	9.13	3.38E−03
	Ran	Sex	0.01	0.01	1	81.77	6.31	1.40E−02	0.02	0.02	1	79.93	1.37	2.45E−01
		Temperature	0.13	0.13	1	73.50	81.17	1.69E−13	0.22	0.22	1	50.76	14.05	4.56E−04
		Sex:Temperature	0.02	0.02	1	81.77	15.23	1.94E−04	0.27	0.27	1	80.00	17.43	7.54E−05
	Cbk	Sex	0.00	0.00	1	82.00	0.43	5.12E−01	0.05	0.05	1	79.99	11.03	1.35E−03
		Temperature	0.70	0.70	1	82.00	90.14	7.49E−15	0.04	0.04	1	65.86	10.21	2.15E−03
		Sex:Temperature	0.01	0.01	1	82.00	1.22	2.73E−01	0.00	0.00	1	80.00	0.02	8.95E−01
	Cpa	Sex	0.01	0.01	1	81.72	1.92	1.69E−01	0.18	0.18	1	79.14	32.02	2.35E−07
		Temperature	0.67	0.67	1	74.17	107.59	4.36E−16	0.01	0.01	1	79.14	1.29	2.60E−01
		Sex:Temperature	0.00	0.00	1	81.70	0.00	9.59E−01	0.07	0.07	1	78.96	11.58	1.05E−03
*D.mojmoj*	Pat	Sex	0.00	0.00	1	23.98	0.12	7.35E−01	0.04	0.04	1	23.02	21.50	1.15E−04
		Temperature	0.11	0.11	1	18.17	10.03	5.29E−03	0.36	0.36	1	23.87	194.32	5.81E−13
		Sex:Temperature	0.00	0.00	1	23.98	0.04	8.40E−01	0.01	0.01	1	23.02	2.89	1.03E−01
	Odk	Sex	0.00	0.00	1	23.55	1.47	2.38E−01	0.04	0.04	1	23.91	14.21	9.45E−04
		Temperature	0.32	0.32	1	5.88	452.42	8.74E−07	0.12	0.12	1	20.88	47.59	8.38E−07
		Sex:Temperature	0.00	0.00	1	23.55	1.72	2.03E−01	0.00	0.00	1	23.91	0.06	8.16E−01
	Ran	Sex	0.00	0.00	1	24.00	0.00	9.91E−01	0.00	0.00	1	23.86	0.12	7.29E−01
		Temperature	0.07	0.07	1	24.00	180.37	1.17E−12	0.00	0.00	1	10.33	0.02	9.02E−01
		Sex:Temperature	0.00	0.00	1	24.00	0.13	7.22E−01	0.13	0.13	1	23.86	12.28	1.84E−03
	Cbk	Sex	0.01	0.01	1	23.00	0.85	3.66E−01	0.00	0.00	1	24.00	0.19	6.69E−01
		Temperature	0.12	0.12	1	3.01	11.53	4.24E−02	0.00	0.00	1	24.00	0.18	6.72E−01
		Sex:Temperature	0.02	0.02	1	23.00	1.50	2.33E−01	0.02	0.02	1	24.00	7.35	1.22E−02
	Cpa	Sex	0.04	0.04	1	22.86	3.47	7.54E−02	0.00	0.00	1	24.00	0.90	3.53E−01
		Temperature	0.19	0.19	1	23.84	16.78	4.18E−04	0.01	0.01	1	24.00	2.14	1.56E−01
		Sex:Temperature	0.09	0.09	1	22.86	8.15	9.00E−03	0.00	0.00	1	24.00	0.11	7.44E−01
*D.rep*	Pat	Sex	0.00	0.00	1	49.00	0.36	5.54E−01	0.00	0.00	1	46.87	2.02	1.62E−01
		Temperature	0.04	0.04	1	49.00	5.95	1.84E−02	0.00	0.00	1	48.34	2.19	1.45E−01
		Sex:Temperature	0.00	0.00	1	49.00	0.00	9.84E−01	0.00	0.00	1	46.02	0.47	4.97E−01
	Odk	Sex	0.00	0.00	1	45.37	0.09	7.70E−01	0.07	0.07	1	49.00	19.57	5.39E−05
		Temperature	0.07	0.07	1	48.26	50.25	5.24E−09	0.00	0.00	1	49.00	0.11	7.40E−01
		Sex:Temperature	0.00	0.00	1	44.19	1.30	2.60E−01	0.01	0.01	1	49.00	3.90	5.40E−02
	Ran	Sex	0.00	0.00	1	48.96	2.31	1.35E−01	0.14	0.14	1	49.00	8.97	4.29E−03
		Temperature	0.04	0.04	1	46.59	87.75	2.75E−12	0.00	0.00	1	49.00	0.00	9.55E−01
		Sex:Temperature	0.00	0.00	1	47.62	2.48	1.22E−01	0.13	0.13	1	49.00	8.48	5.38E−03
	Cbk	Sex	0.06	0.06	1	44.89	7.75	7.85E−03	0.01	0.01	1	48.70	6.80	1.21E−02
		Temperature	0.22	0.22	1	48.09	29.38	1.89E−06	0.00	0.00	1	46.11	0.88	3.53E−01
		Sex:Temperature	0.16	0.16	1	44.61	20.68	4.13E−05	0.00	0.00	1	46.99	0.44	5.10E−01
	Cpa	Sex	0.03	0.03	1	49.00	0.98	3.26E−01	0.00	0.00	1	47.02	0.01	9.27E−01
		Temperature	0.68	0.68	1	45.04	22.07	2.50E−05	0.00	0.00	1	46.46	0.10	7.56E−01
		Sex:Temperature	0.00	0.00	1	46.86	0.03	8.53E−01	0.01	0.01	1	45.29	1.83	1.83E−01

Note

Results of analysis of variance for the effect and interaction of sex and temperature on the different pigmentation traits per body part (model: *lm(Trait* *~ Sex * Temperature + (1|Replicate)*). Exceptionally for *D. simulans,* model: *lm(Trait* *~ Strain * Sex * Temperature + (1|Replicate)*), where *Strain* corresponds to the different genetic backgrounds studied in this species (*D.sim* A and *D.sim* B). Non‐significant effects (*p*‐value >.05) are shown in grey.
