# Corrigendum

**DOI:** 10.1002/ece3.9170

**Published:** 2022-08-02

In the paper published by Lafuente et al. (2021), the below corrections in Section 2.2, Figures 1, 3, 4, and 5, as well as the supplementary tables and figures have been corrected in the article.

- Section 2.2: Where it reads “Dmax‐Dbk,” it should read “(Dmax‐Dbk)/Dmax.”
- Section 2.2: Where it reads “Taking the sequence of normalized darkness values along a transect, we estimated its two enveloping lines,” it should read “From the sequence of averaged RGB pixel values corresponding to each transect, we calculated the Euclidean distance of each pixel's color coordinates to the color white and estimated the enveloping lines of the upper and lower extremes of these values.”
- Figure 1—legend: Where it reads “By calculating the distance between each of those pixels to the black,” it should read “By calculating the distance between each of those pixels and white.”
- Figure 1—panel a: Where it reads “Euclidean distance of each pixel to black” in the y axes, it should read: “Euclidean distance of each pixel to white.”
- Figures 3–5, Figures S1‐S2, and appendix Tables A2‐A5: Where it reads “Cpa,” it should read “Cbk,” and vice‐versa.

The authors apologize for these errors.
