# Supplementary material for: Many ways to make darker flies: Intra‐ and interspecific variation in Drosophila body pigmentation components
Source: Ecol Evol. 2021 May 25;11(12):8136–55. doi: 10.1002/ece3.7646 (PMC8216949; doi:10.1002/ece3.7646)
Supplement: Supplementary file 1 — Fig S1‐2 [file ECE3-11-8136-s001.pdf]

SUPPLEMENTARY FIGURES

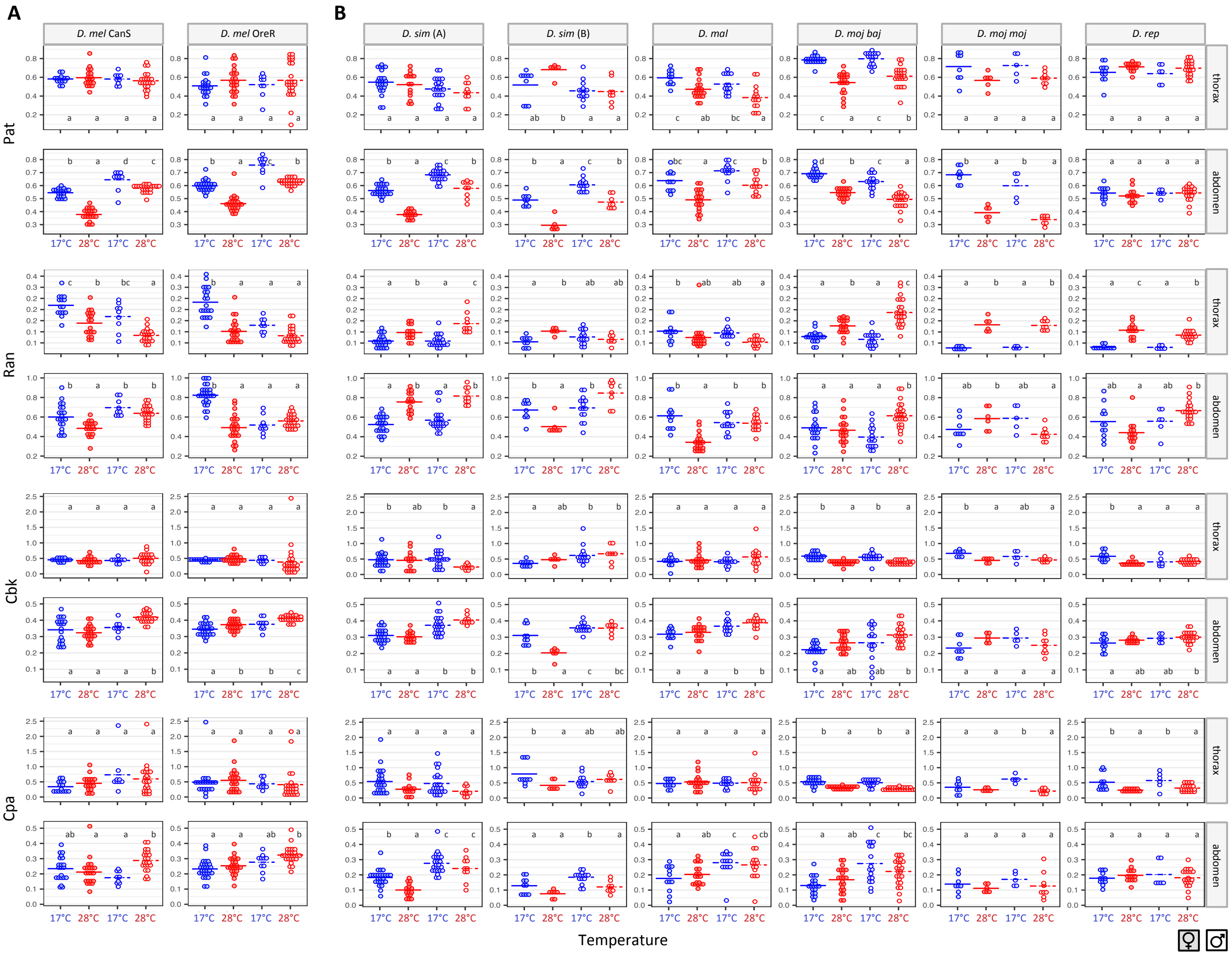

Figure S1. Variation in pigmentation traits with sex and temperature in *Drosophila*. For each population/species, temperature, sex, and body part, dot plots represent variation for pigmentation traits Pat, Ran, Cbk, and Cpa (individual data points and means, represented with bar). Females/males are shown as closed/empty circles and flies reared at 17°C/28°C are shown in blue/red. **A.** *D. melanogaster* laboratory populations. Results of statistical test for the effect of sex, temperature, and their interaction on each of the traits are shown in Table 2. Letters in dot plots indicate results of post-hoc pairwise comparisons between groups: different letters when significantly different (p-value<0.05 for Tukey's honest significance test). **B.** *Drosophila* species. Results of the statistical test for effect of sex, temperature and their interaction are in Table 5. Letters in dot plots indicate results of post-hoc pairwise comparisons between groups: different letters when significantly different (p-value<0.05 for Tukey's honest significance test). For *D. simulans* (*D. sim*), we had two different strains (A and B), which are shown independently in the graph and were included in the statistical model.

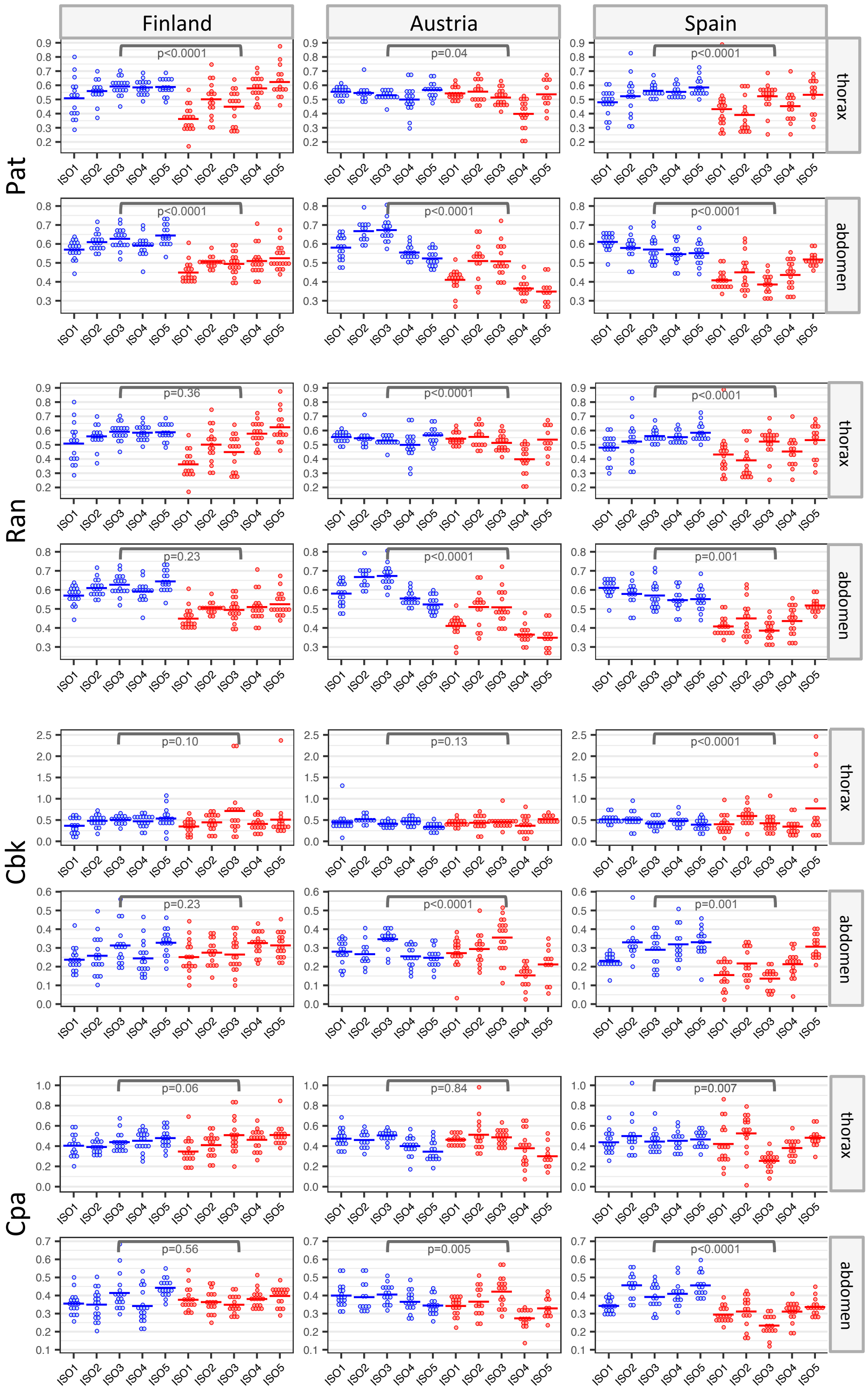

Figure S2. Variation in pigmentation traits in *D. melanogaster* European populations. In each graph, dot plots represent variation for pigmentation traits (individual data points and means, represented with bar) with flies reared at 17°C/28°C are shown in blue/red. For each geographical population we phenotyped females from five genotypes (i.e. isogenic lines). Results of statistical test for the effect of location, genotype and temperature are shown in Table 4. Results of the statistical test (p-value) for the effect of temperature on each of the traits are shown in plots.
